# Amelioration of Hypertriglyceridemia with Hypo-Alpha-Cholesterolemia in LPL Deficient Mice by Hematopoietic Cell-Derived LPL

**DOI:** 10.1371/journal.pone.0025620

**Published:** 2011-09-29

**Authors:** Yinyuan Ding, Ling Zhang, Yuhui Wang, Wei Huang, Yin Tang, Lu Bai, Colin J. D. Ross, Michael R. Hayden, George Liu

**Affiliations:** 1 Institute of Cardiovascular Sciences and Key Laboratory of Molecular Cardiovascular Sciences, Peking University, Beijing, China; 2 Department of Medical Genetics, Centre for Molecular Medicine and Therapeutics, University of British Columbia, Vancouver, British Columbia, Canada; Leiden University Medical Center, The Netherlands

## Abstract

**Background:**

Macrophage-derived lipoprotein lipase (LPL) has been shown uniformly to promote atherosclerotic lesion formation while the extent to which it affects plasma lipid and lipoprotein levels varies in wild-type and hypercholesterolemic mice. It is known that high levels of LPL in the bulk of adipose tissue and skeletal muscle would certainly mask the contribution of macrophage LPL to metabolism of plasma lipoprotein. Therefore, we chose LPL deficient (LPL^-/-^) mice with severe hypertriglyceridemia as an alternative model to assess the role of macrophage LPL in plasma lipoprotein metabolism via bone marrow transplant, through which LPL will be produced mainly by hematopoietic cell-derived macrophages.

**Methods and Results:**

Hypertriglyceridemic LPL^-/-^ mice were lethally irradiated, then transplanted with bone marrow from wild-type (LPL^+/+^) or LPL^-/-^ mice, respectively. Sixteen weeks later, LPL^+/+^ →LPL^-/-^ mice displayed significant reduction in plasma levels of triglyceride and cholesterol (408±44.9 vs. 2.7±0.5×10^3^ and 82.9±7.1 vs. 229.1±30.6 mg/dl, p<0.05, respectively), while a 2.7-fold increase in plasma high density lipoprotein- cholesterol (p<0.01) was observed, compared with LPL^-/-^→LPL^-/-^ control mice. The clearance rate for the oral fat load test in LPL^+/+^ →LPL^-/-^ mice was faster than that in LPL^-/-^→LPL^-/-^ mice, but slower than that in wild-type mice. Liver triglyceride content in LPL^+/+^→LPL^-/-^ mice was also significantly increased, compared with LPL^-/-^→LPL^-/-^ mice (6.8±0.7 vs. 4.6±0.5 mg/g wet tissue, p<0.05, n = 6). However, no significant change was observed in the expression levels of genes involved in hepatic lipid metabolism between the two groups.

**Conclusions:**

Hematopoietic cell-derived LPL could efficiently ameliorate severe hypertriglyceridemia and hypo-alpha-cholesterolemia at the compensation of increased triglyceride content of liver in LPL^-/-^ mice.

## Introduction

Lipoprotein lipase (LPL) is the rate-limiting enzyme responsible for hydrolysis of circulating triglycerides (TGs) carried in very low density lipoprotein (VLDL) and chylomicron (CM). Generally, LPL is produced primarily by muscle and adipose cells. However, a small amount of LPL is also detectable in differentiated macrophages and macrophage- derived foam cells in atherosclerotic lesions [1], [2].

It is well documented that LPL plays a central role in the metabolism of TG-rich lipoproteins (TRLs). Total LPL deficiency in humans (type I hyperlipoproteinemia) is associated with severe hypertriglyceridemia and hypo-alpha-cholesterolemia. Moreover, the major role of muscle LPL in the regulation of plasma TG levels has been determined by studies done in transgenic mice expressing LPL exclusively in skeletal or cardiac muscle [3], [4]. Despite mounting evidence consistently suggesting the proatherosclerotic function of macrophage LPL in atherosclerotic lesion formation in vivo [5], [6], [7], the relative contribution of macrophage LPL to lipoprotein metabolism remains unclear. Through the introduction of LPL gene deleted macrophages by fetal liver cell transplantation into wild-type C57BL/6 or LDL receptor-deficient mice, Babaev et al demonstrated that macrophage LPL deficiency had no effect on serum lipid levels[8], [9]. In contrast, Van Eck et al observed that transplantation of LPL-deficient hematopoietic stem cells into lethally irradiated LPL^+/+^ mice did exert a mild but significant impact on plasma lipids levels with 30% elevation in TG while 8% reduction in total cholesterol (TC) when the animals were fed on normal chow diet. On high fat diet no difference in TG was found between the two bone marrow transplantation (BMT) groups whereas reduction in TC in macrophage-specific LPL deleted mice became more prominent [10]. These studies thus far have provided a valuable but unresolved picture of macrophage LPL in lipoprotein metabolism.

It is obvious that the effects of macrophage LPL on the plasma lipids and lipoproteins would be easily masked by large amounts of LPL synthesized in adipose tissue and skeletal muscle mass. For this reason, the distinct physiological role of macrophage LPL in lipoprotein metabolism might only be properly assessable under conditions in which LPL activity is limiting, such as in LPL^-/-^ recipient mice, as suggested by Van Eck and Babaev et al[8], [9], [10]. However, homozygous LPL knock-out mice die shortly after birth, making it difficult to discern the effects of macrophage LPL from that of the whole-body LPL pool in a well-controlled animal model system[11].

In previous studies, we have reported that gene transfer of the human LPL S447X variant successfully rescued LPL-deficient mice to adulthood [12]. With this specific hypertriglyceridemic (HTG) model, we have determined the effects of LPL deficiency on atherosclerosis development, pancreatitis susceptibility and cognitive function [13], [14], [15]. Recently, Nordestgaard BG et al reported that elevated nonfasting TG levels were associated with increased risk of myocardial infarction, ischemic heart disease and death in men and women [16]. Moreover, hypertriglyceridemia has been increasingly recognized as an independent risk factor in the pathogenesis of atherosclerosis [13], [17], [18]. Therefore, it is of particular interest to investigate whether LPL from macrophages plays any role in the LPL deficient severe HTG state, which may provide new insight on the effects of macrophage LPL on lipoprotein metabolism other than atherosclerosis.

In the current study, bone marrow from wild-type (LPL^+/+^) or LPL deficient (LPL^-/-^) mice was transplanted into irradiated LPL^-/-^ mice as an alternative model, in which LPL will be produced mainly from hematopoietic cell-derived macrophages. It was interesting to note that hematopoietic cell-derived LPL alone was nearly sufficient to normalize the lipoprotein metabolism at the compensation of increased TG content of liver in LPL^-/-^ mice. Moreover, these results provided direct evidence that LPL derived from hematopoietic cell also performs a crucial role in clearance of TRLs in the setting of severe hypertriglyceridemia.

## Results

### Plasma lipid and lipoproteins

**Figure 1:**
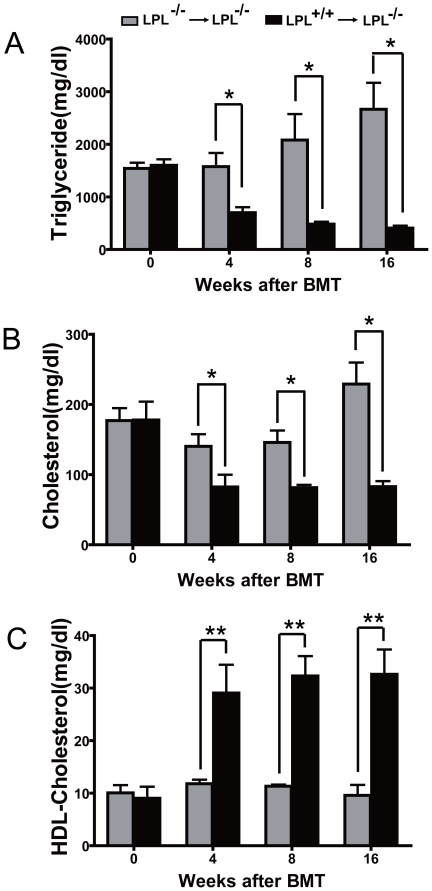
Effects of hematopoietic cell-derived LPL on plasma lipid levels. All mice were fasted overnight and plasma at 0, 4, 8 and 16 weeks after BMT was collected. Plasma TG levels (A), TC levels (B) and HDL-C levels (C) were measured as described in methods sections. Values were expressed as means ± SEM. * p<0.05, ** p<0.01 for LPL^+/+^→LPL^-/-^ group compared to LPL^-/-^ →LPL^-/-^ group.

All mice were fed a normal chow diet and fasted plasma. TG, TC and HDL cholesterol (HDL-C) levels were measured at the time of BMT up to 16 weeks. As early as 4 weeks after BMT, plasma TG and TC levels in LPL^+/+^→LPL^-/-^ mice were rapidly reduced by 59.4% (699.6±105.1 vs. 1.7±0.2×10^3^ mg/dl, p<0.05) and 46.3% (82.4±17.4 vs. 153.5±16 mg/dl, p<0.05), while an almost 3-fold increase in plasma HDL-C (29.1±5.4 vs. 11.8±0.5 mg/dl, p<0.01) was observed, compared with LPL^-/-^→LPL^-/-^ control mice (figure 1A,1B,1C). The disparity between the two groups was further enlarged at the time of 8 and 16 weeks after BMT.

**Figure 2:**
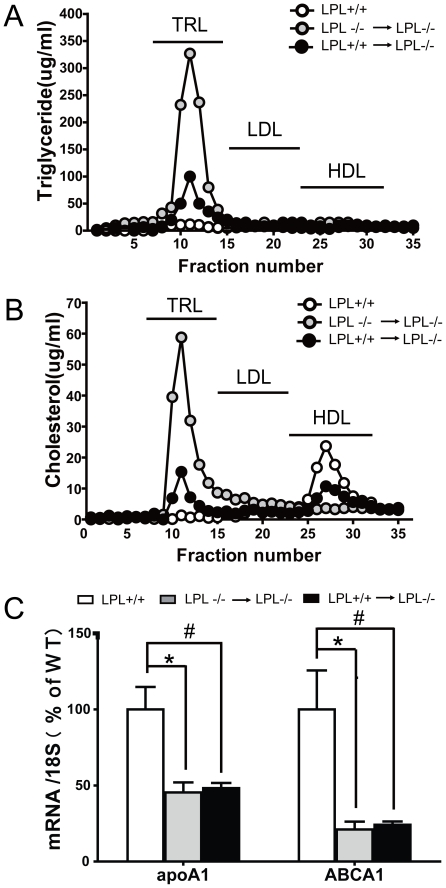
Plasma lipoprotein particle characterized by FPLC and hepatic gene expression involved in HDL synthesis. Lipoprotein profiles were determined by FPLC followed by TG (A) and cholesterol (B) analysis of each fraction. C: The expression levels of apoA1 and ABCA1 were determined by the RT-PCR methods. ^#^ p<0.05, * p<0.05, for LPL^+/+^→LPL^-/-^ and LPL^-/-^ →LPL^-/-^ mice vs. LPL^+/+^ mice, respectively (n = 6 for each group).

The lipoprotein profiles are shown in figures 2A and 2B, as expressed either in TG or cholesterol (Chol.) contents in each fraction analyzed by fast-performance liquid chromatography (FPLC) of fasting plasma for normal non-BMT transplanted (LPL^+/+^), LPL^-/-^→LPL^-/-^ and LPL^+/+^→LPL^-/-^ mice at 20 weeks after BMT. Consistent with the results of plasma lipid level measurements, LPL^+/+^→LPL^-/-^ mice showed a marked reduction of TG and Chol. content distributed in CM/VLDL fractions and a moderate increased HDL-C concentrations. In light of the crucial roles of apoA1 and ABCA1 in the formation of mature HDL particles, then the expression levels of those genes in liver tissues were examined. As shown in figure 2C, no significant changes at expression levels were observed between the two BMT groups, whereas their levels were significantly lower than those of wild-type mice (p<0.05).

**Figure 3:**
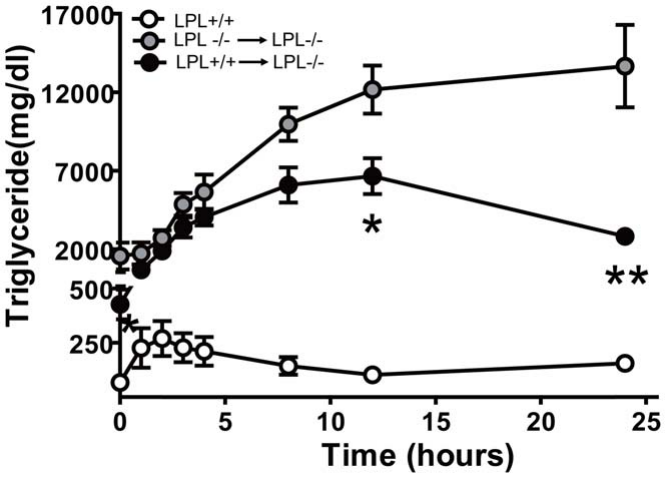
Effects of hematopoietic cell-derived LPL on clearance of intragastric fat load. Each group mice were received an oral fat load test and plasma TG levels were determined at the respective time points. Values were expressed as means ± SEM. * p<0.05, ** p<0.01, for LPL^+/+^→LPL^-/-^ mice compared to LPL^-/-^ → LPL^-/-^ group, respectively.

Subsequently, an oral fat tolerance test was used to assess the catabolism of both enterogenous and liver-derived TRLs. Figure 3 shows that plasma TG levels peaked during 2 h and 12 h in LPL^+/+^ and LPL^+/+^→LPL^-/-^ mice, with peak values of TG at 270±80.4 mg/dl and 6.6±1.1×10^3^ mg/dl, respectively. However, plasma TG levels in LPL^-/-^→LPL^-/-^ mice was increased constantly from initial levels around 1.6×10^3^ mg/dl to near 14×10^3^ mg/dl without discernable peak during a 24 h observing period, with higher TG levels than those in LPL^+/+^→LPL^-/-^ mice at time points of 12 h (p<0.05) and 24 h (p<0.01). Thus, the clearance of plasma exogenous TG, mainly carried in CMs was accelerated in LPL^+/+^→LPL^-/-^ mice, although it is still much slower than that in LPL^+/+^ mice.

### LPL activity and LPL expression

**Figure 4:**
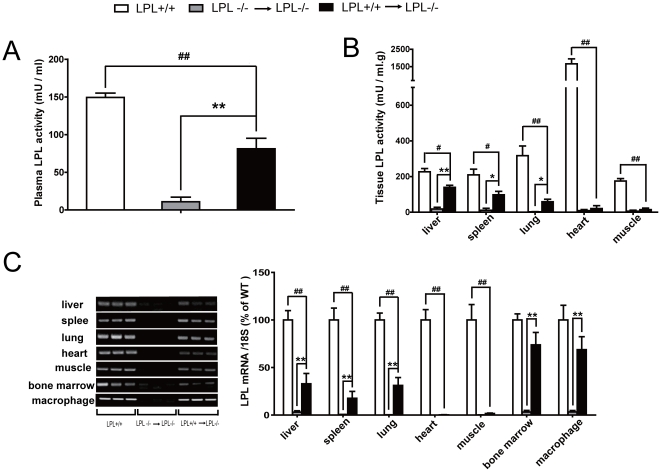
Effects of hematopoietic cell-derived LPL on LPL activity and LPL expression. A: Post-heparin LPL enzyme activity assayed as described earlier. B: LPL activity of various tissues was measured. C: LPL gene expression in various tissues and cells was determined by RT-PCR (left panel) or Quantitative Real-Time PCR (right panel) method. Columns and bars represent means±SEM. * p<0.05, ** p<0.01, for LPL^+/+^→LPL^-/-^ mice vs. LPL^-/-^ → LPL^-/-^ mice; ^#^ p<0.05, ^# #^ p<0.01, for LPL^+/+^→LPL^-/-^ mice vs. LPL^+/+^ mice (n = 6 for each group).

**Figure 5:**
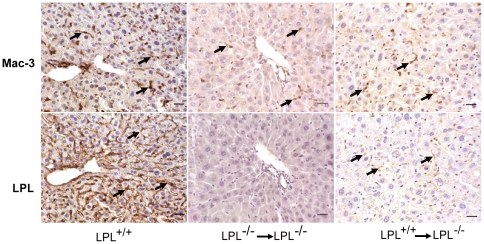
Immunocytochemical detection of macrophage and LPL staining in wild-type, LPL^-/-^ mice transplanted with bone marrow from wild-type or LPL^-/-^ mice, appeared in brown. Representative images show macrophages (upper panel) and LPL protein (lower panel) stain in paraffin sections of liver. Bars represent 20 µm.

To determine whether the reduced plasma TG levels and accelerated fat clearance was due to the effect of hematopoietic cell-derived LPL in LPL^+/+^→LPL^-/-^ mice, LPL activity and LPL mRNA levels were compared among three groups. As shown in figure 4A, fasted post-heparin plasma LPL activities in LPL^+/+^→LPL^-/-^ mice increased by 12-fold (71.7±7.8 vs. 5.5±3.7 mU/ml, p<0.01) compared with LPL^-/-^→LPL^-/-^ mice, which were still significantly lower than that in wild-type mice (140±9.6 mU/ml, p<0.01). Substantial LPL activity and expression were predominantly found in macrophage-enriched organs such as the liver, spleen and lung in LPL^+/+^→LPL^-/-^ mice whereas similar LPL activity and expression was absent in LPL^-/-^→LPL^-/-^ mice. However, LPL activity and expression in tissues were significantly lower in LPL^+/+^→LPL^-/-^ mice than those of wild-type mice (figure 4B and 4C). In addition, no human Ad-LPLS447X gene expression was found in any groups of mice (data not shown). Similar to macrophage, LPL protein in LPL^+/+^ and LPL^+/+^→LPL^-/-^ mice was stained positively and localized close to trabecular veins and sinusoidal space in tissue sections of the liver, whereas no LPL positive staining was found in LPL^-/-^→LPL^-/-^ mice (figure 5).

### Histological and Lipid Analysis in Liver

**Figure 6:**
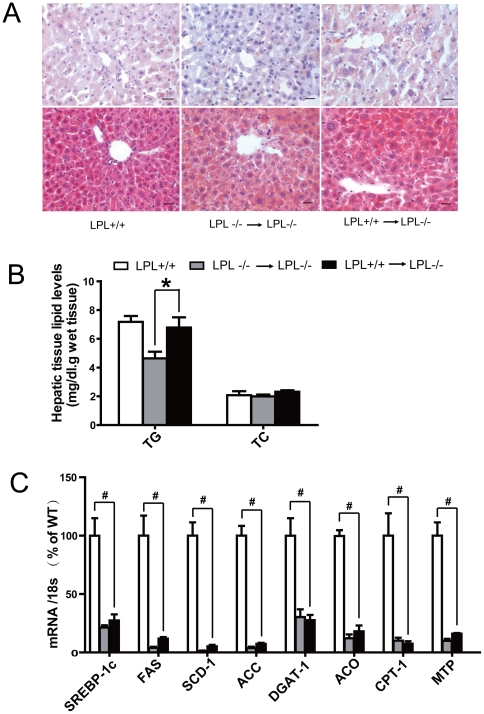
Effects of hematopoietic cell-derived LPL on hepatic histology and lipid content. A: Images show Oil Red O (upper panel) and Hematoxylin-eosin stain (lower panel) in cryocut sections of liver. Bars represent 20 µm. B: Total lipids were extracted from livers of mice. TG and TC contents in each group were determined as described above. C: Messenger RNA levels of hepatic lipid metabolism involved gene. Columns and bars represent means±SEM. ^#^ p<0.05, for LPL^+/+^→LPL^-/-^ mice vs. LPL^+/+^ mice; * p<0.05, for LPL^+/+^→LPL^-/-^ mice vs. LPL^-/-^ → LPL^-/-^ mice (n = 6 for each group).

As shown in figure 6A, LPL^-/-^→LPL^-/-^mice exhibited normal liver architecture but less intracellular lipid vacuoles, compared with LPL^+/+^ and LPL^+/+^→LPL^-/-^ mice. Liver TG levels in LPL^+/+^→LPL^-/-^ mice were increased by 47.8% when compared with that in LPL^-/-^→LPL^-/-^mice (6.8± 0.7 vs. 4.6±0.5 mg/g wet tissue, p<0.05), identical to that in LPL^+/+^ mice (7.2±0.4 mg/g wet tissue, p>0.05) (figure 6B). No significant changes were observed in the expression levels of those genes related to hepatic lipid metabolism between the two BMT groups, which were markedly lower than those of wild-type mice (p<0.01) (figure 6C).

## Discussion

In this study, through transplantation of wild-type hematopoietic stem cells, LPL activity and mRNA were noticeably detected in macrophage-enriched organs in LPL^-/-^ mice, indicating that LPL^-/-^ chimeric mice expressing LPL exclusively in hematopoietic cell were successfully generated through BMT. Given that macrophages are the only leukocytes that express LPL, LPL^+/+^→LPL^-/-^ mice would undoubtly serve as an alternative model to study the role of macrophage-derived LPL in lipoprotein metabolism.

Our results clearly showed for the first time that hematopoietic cell-derived LPL alone was nearly sufficient to compensate for the lack of LPL in other tissues with regard to improving hypertriglyceridemia. Sixteen weeks after BMT, LPL^+/+^→LPL^-/-^ mice were observed up to 12-fold increase of plasma LPL catalytic activity, a significant increase in plasma HDL-C and rapid declines of TG and TC levels, characterized by drastically decreased TG and Chol. content in the VLDL/CM lipoprotein fractions. These findings are in contrast to previous studies using mice with normal LPL function as recipients in BMT experiments, in which macrophage LPL exerted no or mild effects on plasma lipid levels[8], [9], [10]. However, in a study on the functional role of cardiac LPL, Levak-Frank et al demonstrated the importance of choosing proper animal models in assessing the contribution of LPL from various tissues to lipoprotein metabolism [3]. While heart-specific LPL expression in wild-type mice only reduced plasma TG to 50% of normal controls, the similar expression of LPL in homozygous LPL deficient mice rescued the neonatal lethality and completely prevented the extreme HTG phenotype which could have occurred in LPL deficient mice if these animals had survived to adulthood. As a result, the effect of LPL derived from bone marrow was made discernable in a LPL-deficient background, which otherwise would be masked by high levels of LPL present in adipose tissue and skeletal muscle. In fact, our findings extended the observations made earlier by Hussain et al who found that guinea pigs, dogs and rats did not exhibit significant uptake of exogenous CMs by bone marrow whereas rabbit and a New World primate accumulated 1/2 and 1/10 of injected CMs in bone marrow, respectively[19], [20]. The current results implies that hematopoietic cell-derived LPL rather than bone marrow in mice may participate actively in CM metabolism and promote the removal of plasma TGs carried in CMs in normal conditions.

Despite markedly reduced plasma TG levels by introduction of LPL via BMT, the acute plasma fat clearance rate in LPL^+/+^ →LPL^-/-^ mice was still significantly slower than that in wild-type mice, indicating that hematopoietic cell-derived LPL activity alone is nevertheless inadequate for complete correction of severely impaired fat tolerance in LPL^-/-^ mice.. Regarding this, it appears to serve as a secondary determinant of plasma TG hydrolysis unlike muscle and adipose tissue-derived LPL.

In addition to rapidly reduced TG levels, plasma HDL-C levels were also increased by 1.7-fold in LPL^+/+^→LPL^-/-^ mice during the same duration. Our results provided strong evidence that hematopoietic cell-derived LPL could accelerate the clearance of plasma atherogenic lipoproteins such as CM/VLDL remnant in the HTG state, and lead to the generation of anti-atherogenic lipoproteins, HDLs. However, we did not find the up-regulation of apoA1 and ABCA1 expression in the liver, the 2 of the major factors involved in HDL biogenesis. In fact, it is known that HDL can also be generated via enzymatic action of LPL, ie, lipolysis of TRLs [21]. In this process, HDL will be formed mainly through the transfer of surface components from TG-rich lipoproteins, ie, phospholipids and apoproteins like apoAI, apoCs. Therefore, when LPL activity is increased HDL could be generated without up-regulation of apoAI and ABCA1 gene expression. In the present study it is conceivable that lipolysis of TRLs mediated by LPL derived from hematopoietic cell in LPL^-/-^ mice contributes directly to the generation and maturation of HDL particles through direct transfer of surface components from TG-rich lipoprotein catabolism. To this end, our findings are in good agreement with earlier study by Strauss GJ et al, who reported that LPL, together with apoA1 and ABCA1, was crucial in the formation of mature HDL particles [22]. However, direct evidence may be obtained in further studies by generating ABCA1/LPL or apoA1/LPL double-knockout mice.

It is also noticeable in current study that introduction of LPL-producing hematopoietic cells to LPL^+/+^→LPL^-/-^ mice resulted in an increased liver TG content as well, similar to the levels found in normal mice. This could be related to the enhanced expression of LPL in the liver after BMT. Catalytically active LPL in the liver will certainly increase the local generation of free fatty acids via hydrolysis of TG carried in CM/VLDL in the hepatic circulation, combined with pre-existing FFA pool as demonstrated by Teusink B et al [23]. Influx of free fatty acids into hepatocytes will results in TG synthesis for storage or VLDL package/secretion. However, no significant change of the hepatic genes involved in TG synthesis and VLDL package/secretion could be observed between the mice receiving either LPL^+/+^ or LPL^-/-^ hematopoietic cells. Therefore, the major source to accumulated hepatic TG in LPL^+/+^→LPL^-/-^ mice could be from direct uptake of remnant TG-rich lipoproteins during their catabolism processing by LPL in the liver because these remnant lipoproteins will easily pass through the liver fenestrae and become accessible to receptors for remnant uptake. This notion has received support from a study which demonstrated that liver-specific LPL transgenic expression in LPL knockout mice led to excessive amounts of intracellular lipid droplets in the liver [24].

In a previous study[13],we reported the occurrence of spontaneous atherosclerosis in LPL deficient HTG mice older than 15 months,which was further confirmed by a recent report on atherogenicity in Gpihbp1-deficient HTG mice[18]. In light of the proatherosclerosis role of macrophage LPL, this raises an issue of whether or not macrophage LPL expressing under the hypertriglyceridemia state further increases the susceptibility of atherosclerosis in LPL^-/-^ mice. Surprisingly, no obvious atherosclerotic plaques were observed in both BMT and wild-type mice in our current study (data not shown). Considering the limited life expectancy of LPL deficient mice undergoing BMT, it would be difficult to perform atherogenic studies in these mice. It might be helpful to generate LPL and apoE double-knockout mice with combined severe hypercholesterolemia and hypertriglyceridemia in further determining the correlation between the more favorable lipoprotein profile resulting from LPL expression in hematopoietic cell and risk of atherosclerosis in vivo.

In conclusion, our results show that hematopoietic cell-derived LPL could ameliorate severe hypertriglyceridemia and hypo-alpha-cholesterolemia, as well as normalize hepatic lipid content even when LPL is absent in muscle and adipose tissue in LPL^-/-^ mice. This study should shed light on the special role that LPL derived from hematopoietic cells in lipoprotein metabolism and atherosclerosis.

## Materials and Methods

### Animals

LPL-deficient mice were rescued from neonatal death by intramuscular injection of a recombinant adenovirus coding for human LPL beneficial mutant, Ad-LPLS447X, as previously described [12], [13]. Genotyping was done using genomic DNA extracted from tails by PCR and was designated LPL^-/-^ and LPL^+/+^ accordingly.

All mice used for BMT experiments were maintained in microisolator cages with sterilized regular chow and had free access to autoclaved acidified water (100 mg/L neomycin and 10 mg/L polymyxin B sulfate). All procedures were performed in accordance with the "Principles of Laboratory Animal Care" and approved by the Animal Care Committee, Peking University Health Science Center (No.La2010-061).

### Irradiation and BMT

Female LPL^-/-^ C57BL/6 mice (aged 6 to 8 weeks) were subjected to 900 rads lethally total body irradiation by using a cesium gamma source. Bone marrow cells used for repopulation were extracted from the femur and tibia of male LPL^-/-^ C57BL/6 mice and male LPL^+/+^ C57BL/6 mice (aged 6 weeks). Four hours later, 5×10^6^ bone marrow cells in 300 µl of RPMI-1640 media from either male LPL^-/-^ or male LPL^+/+^ mice were injected into the tail vein of all irradiated mice. Age-matched female LPL^+/+^ littermates that did not undergo BMT served as control group.

All mice were sacrificed and blood samples were collected for FPLC or LPL activity assay at the end of the twentieth week after BMT, followed by the removal of liver, spleen, lung, heart and skeletal muscle. The tissues were processed for the measurement of lipid content, RNA and tissue LPL enzyme activity. Resident peritoneal macrophages in mice were harvested by washing the peritoneal cavity with RPMI 1640 after intraperitoneal injection of 2 ml 3% thioglycollate broth 4 days ago[6]. Bone marrow cells were collected as described above and prepared with macrophages for RNA extraction.

### Lipid Analysis in Plasma and Liver

For lipid analysis, blood samples were collected by retro-orbital bleeding under anesthesia following an overnight fast for the determination of TC and TG levels using commercial kits (Sigma, MO, USA). HDL-C was quantified after precipitation of apolipoprotein B-containing lipoproteins with an equal volume of a 20% polyethylene glycol solution, as described previously [25]. For the analysis of the plasma lipoprotein distribution, lipoproteins in 200 µl of pooled plasma from 3 mice per group were isolated by FPLC using a Superose 6 column (Amersham Bioscience Inc., USA) as described [10]. Thirty five fractions of 0.5 ml each were collected and enzymatically assayed for Chol. and TG content.

### Fat Tolerance Test

Mice were fasted overnight. After taking a basal blood sample by retro-orbital bleeding at t = 0, animals received an intragastric load of 200 µl olive oil gavage. Additional blood samples were drawn 1, 2, 3, 4, 8, 12 and 24 h after olive oil administration. Plasma TG levels were measured as described above.

### Determination of LPL Enzyme Activity

To determine plasma LPL activity, post-heparin plasma was obtained as described above and LPL activity was determined using radioactive trioleoylglycerol emulsion substrate according to an established method[7]. Enzyme activity was expressed as mU/ml (1 mU corresponds to 1 nM free fatty acid generated per minute).

For LPL activity of tissues, individual tissues were weighed and then homogenized in 25 mM ammonia buffer prepared. The homogenates were centrifuged at 10×10^3^ g for 20 min at 4°C and the clear supernatant was used for the assay of LPL activity. Tissue LPL enzyme activity was expressed as mU/ml.g wet tissue.

### RNA Isolation and Real-Time PCR

Total RNA from tissues was isolated using Tri Reagent (MRC, USA) and first-strand cDNA was generated by using a RT kit (Invitrogen, USA). Quantitative Real-Time PCR was performed using following primer sets: LPL (5′-GTGGCCGAGAGCGAGAACAT-3′, 5′-GC TTTCACTCGGATCCTCTC-3′); ACC (5′-CCAGACCCTTTCTTCAGC-3′, 5′-TTGTCGTAGTGGCCGTTC-3′); FAS(5′-GCCTCCGTGGACCTTATC-3′, 5′-ACAGACACCTTCCCGTCA -3′); SREBP-1c (5′-AACGTCACTTCCAGCTAGAC-3′, 5′-CCACTAAGGTGCCTACAGAG C-3′); SCD-1 (5′-CGTCAGCACCTTCTTGAGATAC-3′, 5′-TCACTGGCAGAGTAGTCG TAGG-3′); ACO (5′-GGTGGGTGGTATGGTGTCGTAC-3′, 5′-CAAAGACCTTAACGGT CACGTAGTG-3′); DGAT-1 (5′-ATCTGAGGTGCCATCGTC-3′, 5′-ATGCCATACTTGATA AGGTTCT-3′); CPT-1 (5′-CGCACGGAAGGAAAATGG-3′, 5′-TGTGCCCAATATTCCTG G-3′); MTP (5′-GGAAAGCAGAGCGGAGAC-3′, 5′-AGAGCAAGGGTCAGGCAC-3′); apoA1 (5′-GAGGAGCAAGGTATCGG-3′, 5′-GGGAAACAGCCCAGTC-3′); ABCA1 (5′- CGTTTCCGGGAAGTGTCCTA-3′, 5′-GCTAGAGATGACAAGGAGGATGGA-3′) and 18 s (5′-GGAAGTGCACCACCAGGAGT-3′, 5′-TGCAGCCCCGGACATCTAAG-3′). Amplifi- cations were performed in 35 cycles using an opticon continuous fluorescence detection system (MJ Research) with SYBR green fluorescence (Molecular Probes, Eugene, USA). All samples were quantitated for relative quantitation of gene expression by using 18 s ribosomal RNA as an internal standard, then normalized to wild-type.

### Immunohistochemistry

To detection of LPL in liver and heart, liver samples fixed in 4% buffered formalin were embedded in paraffin. Seven-µm-thick sections immersed in 0.01M PBS (PH7.4), and then incubated overnight at 4°C with anti-LPL mAb AM4(recognizes both human and mouse LPL)at 1∶100 dilution after treatment with blocking reagents (Histomouse Kit, ZyMed Co) [26]. The sections were treated with goat HRP-labeled antibodies (Zhongshan Bio Co, China) to mouse IgG at 1∶200 dilution for 45 minutes at 37°C. A SABC kit and DAB substrate were then used to yield a brown reaction product. Slides were counterstained with hematoxylin, mounted with glycerol gelatin (Sigma, MO, USA) and examined with a light microscope.

For the localization of mouse macrophages, a monoclonal rat anti-Mac-3 antibody (BD Pharmingen) was used for the staining of Mac-3 as previously described [13].

### Histological and Lipid Analysis in Liver

For lipid staining, segments of hepatic tissues were embedded in OCT and snap-frozen in liquid nitrogen. Seven-µm-thick cryocut sections were stained with Oil red-O (ORO) or hematoxylin-eosin staining. Lipids of liver tissues were measured by a modification of the method described before [27]. Total lipid were evaporated under nitrogen to dryness and redissolved in 0.5 ml 3% triton X-100 to determine TG and TC.

### Statistical Analysis

Quantitative data were given as means ± SEM. Statistical significance was tested using two-tailed Student's t test and one-way ANOVA (Newman – Keuls test) by the computer program Prism (GraphPad Software). Differences were considered significant at p<0.05.
